# Antitumor effects of intracranial injection of B7-H3-targeted Car-T and Car-Nk cells in a patient-derived glioblastoma xenograft model

**DOI:** 10.1007/s00262-024-03808-0

**Published:** 2024-10-05

**Authors:** Tetsuro Tachi, Noriyuki Kijima, Hideki Kuroda, Syunya Ikeda, Koki Murakami, Tomoyoshi Nakagawa, Moto Yaga, Kanji Nakagawa, Reina Utsugi, Ryuichi Hirayama, Yoshiko Okita, Naoki Kagawa, Haruhiko Kishima, Chihaya Imai, Naoki Hosen

**Affiliations:** 1 Department of Neurosurgery, Graduate School of Medicine, Osaka University, 2-2 Yamadaoka, Suita, Osaka 5650871 Japan; 2 World Premier International Immunology Frontier Research Center, Osaka University, Suita, Osaka Japan; 3 Department of Respiratory Medicine, Osaka General Hospital, Osaka, Japan; 4 Department of Hematology and Oncology, Graduate School of Medicine, Osaka University, 2-2 Yamadaoka, Suita, Osaka 5650871 Japan; 5 Department of Pediatrics, Faculty of Medicine, University of Toyama, Toyama, Japan

**Keywords:** Antitumor effect, CAR-NK cell therapy, Glioblastoma (GBM), B7-H3

## Abstract

**Background:**

Glioblastoma multiforme (GBM) is the most lethal primary brain tumor for which novel therapies are needed. Recently, chimeric antigen receptor (CAR) T cell therapy has been shown to be effective against GBM, but it is a personalized medicine and requires high cost and long time for the cell production. CAR-transduced natural killer (NK) cells can be used for "off-the-shelf" cellular immunotherapy because they do not induce graft-versus-host disease. Therefore, we aimed to analyze the anti-GBM effect of CAR-T or NK cells targeting B7-H3, which is known to be highly expressed in GBM.

**Methods:**

CAR-T cells targeting B7-H3 were generated using previously reported anti-B7-H3 scFv sequences. Cord blood (CB)-derived NK cells transduced with the B7-H3 CAR were also generated. Their anti-GBM effect was analyzed in vitro. The antitumor effect of intracranial injection of the B7-H3 CAR-T or NK cells was investigated in an in vivo xenograft model with patient-derived GBM cells.

**Results:**

Both B7-H3 CAR-T cells and CAR-NK cells exhibited marked cytotoxicity against patient-derived GBM cells in vitro. Furthermore, intracranial injection of CAR-T cells and CAR-NK cells targeting B7-H3 resulted in a significant antitumor effect against patient-derived GBM xenografts.

**Conclusion:**

Not only CAR-T cells but also CB-derived CAR-NK cells targeting B7-H3 may have the potential to eliminate GBM cells.

## Introduction

Glioblastoma multiforme (GBM) is one of the most deadly primary brain tumors, with an overall survival rate of approximately 15–20 months despite standard treatments such as surgery, radiotherapy, and chemotherapy [1]. This highlights the urgent need for novel therapies to improve patient outcomes. Chimeric antigen receptor (CAR) T cell therapy for brain tumors is being investigated extensively and has demonstrated clinical efficacy in early phase clinical trials [2]. Targets such as interleukin-13 receptor alpha 2 (IL13Rα2), epidermal growth factor receptor variant III (EGFRvIII), human epidermal growth factor receptor 2 (HER2), GD2, CD70, CD147, EphA2, and B7-H3 are being investigated in clinical trials [2–8]. In particular, B7-H3 is considered an ideal target for CAR-T cell therapy, as it is highly expressed in over 70% of GBM samples [9, 10] but not in normal brain tissue [11]. Several CAR-T cells targeting B7-H3 have been developed [12–15] and are undergoing clinical trials.

Despite its potential, CAR-T cell therapy faces challenges such as high cost and long cell production time. Unlike T cells, natural killer (NK) cells do not induce graft-versus-host disease (GVHD) when infused into allogeneic donors and are not associated with cytokine release syndrome or immune effector cell-associated neurotoxicity syndrome, which are adverse effects of CAR-T cell therapy [16, 17]. CAR-NK cell therapy targeting CD19 has been shown to be clinically effective in studies of B-cell malignancies [18, 19]. Several preclinical studies and one clinical trial have been conducted on CAR-NK cell therapy against GBM [20]. NK92 cell lines transduced with CARs recognizing EGFRvIII [21, 22], HER2 [20, 23, 24], and B7-H3 [25] have demonstrated efficacy in preclinical GBM models. Human peripheral blood-derived CAR-NK cells targeting EGFRvIII or CD73 and GD2 have shown efficacy in GBM xenograft models [26, 27]. CB-derived CAR-NK cells targeting B7-H3 have shown in vitro cytotoxicity against GBM cells [28]. In this study, we aimed to develop CB-derived CAR-NK cells targeting B7-H3 and investigate their in vivo antitumor effects after intracranial injection into immunodeficient mice engrafted with patient-derived GBM cells.

## Material and Methods

### Cell Lines and Cord Blood Cells

The U87MG cell lines (RRID: CVCL_0022) were purchased from the American Type Culture Collection (Manassas, VA, USA). These cells were cultured in DMEM high glucose media (Thermo Fisher Scientific, Waltham, MA, USA), supplemented with 10% fetal bovine serum (FBS). Patient-derived tumor cell lines were established and maintained in a serum-free culture medium that included epidermal growth factor (EGF) and basic fibroblast growth factor, as detailed in our previous study [9].

Cord blood (CB) cells were sourced from the Kinki Cord Blood Bank and Hyogo Cord Blood Bank, after obtaining informed consent. The conduct of this study was approved by the institutional review boards of Osaka University Graduate School of Medicine, Kinki Cord Blood Bank, and Hyogo Cord Blood Bank.

## Animal Experiments

Six-week-old male NOD/Shi-scid IL2Rγnull (NOG) mice were purchased from the Central Institute for Experimental Animals, Kawasaki, Japan. The conduct of animal experiments was sanctioned by the Institutional Animal Care and Use Committee at Osaka University Medical School (Approval numbers 03–071-000 and 04–028-002). All animal-related procedures were performed in strict adherence to the guidelines of the Animal Experiment Committee at Osaka University.

## Flow Cytometry and Sorting

To assess the expression of B7-H3 on the surface of the target cell line, cells were stained with an anti-B7-H3 antibody (MIH42; BioLegend, San Diego, CA, USA, RRID: AB_10720987) in phosphate-buffered saline supplemented with 1% FBS at 4 °C for 30 min. The cells were then washed and incubated with a PE-conjugated goat antimouse IgG secondary antibody (Poly4053; BioLegend, RRID: AB_315010) at 4 °C for an additional 30 min. Following another washing step, the cells were analyzed using a BD FACS Canto II, BD FACS Celesta, or FACS Aria II flow cytometer (BD Biosciences, Franklin Lakes, NJ, USA). Flow cytometry data were processed using the FlowJo software (BD Biosciences, RRID: SCR_008520). The antibodies utilized for staining included anti-CD3 Cy7PE (UCHT1; BioLegend, RRID: AB_439781), anti-CD56 PE (HCD56; BioLegend, RRID: AB_604101), and goat antihuman F(ab')2 Alexa Fluor 647 (109–607-003; Jackson ImmunoResearch, West Grove, PA, USA, RRID: AB_2337903). Cetuximab (Merck Biopharma, Darmstadt, Germany), targeting the epidermal growth factor receptor (EGFR), was biotinylated using the Biotin Labeling Kit (Dojindo, Kumamoto, Japan) for staining purposes.

## Generation of B7-H3-Knockout (KO) U87MG Cells

We established B7-H3-KO U87MG cells using the CRISPR-Cas9 system. crRNA was synthesized using the design tool from Integrated DNA Technologies (IDT, Coralville, IA, USA). The selected target sequence was AGTGCCACCACTGGGTCTTC. A ribonucleoprotein (RNP) complex was prepared by combining crRNA, tracrRNA (IDT, catalog no. 1072533), and TrueCut Cas9 protein V2 (Thermo Fisher Scientific). This RNP complex was then electroporated into U87MG cells (5 × 10^6^) using the NEPA 21 electroporator (Nepa Gene, Ichikawa, Japan) [29]. Cells devoid of B7-H3 expression were isolated using FACS.

## Development of Chimeric Antigen T Cells targeting B7-H3

The anti-B7-H3 chimeric antigen receptor (B7-H3 CAR) was developed using the anti-B7-H3 single-chain variable fragment (scFv) BRCA84D (MG27A; US patent #8,802,091 B2) from MacroGenics Inc. (Rockville, MD, USA). As a control, we also created CAR-T cells targeting CD19 using the reported sequences of an anti-CD19 monoclonal antibody [30]. The isolated cDNAs for the variable regions of the κ light chain and heavy chain were combined with the transmembrane domain of CD8α, cytoplasmic domains of CD28 and CD3ζ, T2A peptides, and truncated EGFR sequence through overlapping PCR [31]. The B7-H3 CAR construct was then inserted into a retroviral vector. To produce viral supernatants, 293 T cells (RRID: CVCL_0063) were co-transfected with the retroviral vector gag-pol and VSV-G envelope plasmids using Lipofectamine 2000 reagent (Thermo Fisher Scientific). Supernatants containing the retrovirus were collected after 48 and 72 h.

Activated T cells were then subjected to retroviral transduction with the B7-H3 CAR construct. Peripheral blood mononuclear cells (PBMCs) from a single donor were initially activated using anti-CD3 (OKT3; eBioscience, San Diego, CA, USA; RRID: AB_468854) and anti-CD28 (CD28.2; eBioscience; RRID: AB_468926) antibodies and cultured in X-VIVO 15 medium (LONZA, Basel, Switzerland) supplemented with 5% human serum AB (GeminiBio, West Sacramento, CA, USA). On the following day, recombinant human IL-2 (Shionogi Pharma, Osaka, Japan) was added to the cultures at a final concentration of 100 IU/mL. Cells were harvested 2 days post-activation and subjected to retroviral transduction using RetroNectin (Takara Bio, Kusatsu, Japan). Non-treated 48 well plates were coated with 20 μg/ml RetroNectin. The retroviral titer was 3.5 × 10^6^ cfu/ml.

After transduction, the cells were maintained in culture medium with 100 IU/mL IL-2 for 7 days. The efficiency of CAR transduction was assessed by staining the cells with a biotin-conjugated anti-tEGFR antibody and streptavidin-PE (BioLegend).

## Development of CAR-NK Cells Targeting B7-H3 (B7-H3 CAR-NK cells)

K562 cells, engineered to express membrane-bound IL-15 and 4-1BB ligand (K562-mb15-41BBL cells; RRID: CVCL_C7IM), were provided by St. Jude Children’s Research Hospital [32]. The cDNAs encoding the B7-H3 variable regions were linked with CD28 and CD3ζ cDNAs through overlapping PCR. Additionally, T2A-IL-15 cDNA was integrated into the CAR construct. T cells were eliminated using CD3 MicroBeads (Miltenyi Biotec, Bergisch Gladbach, Germany). The T cell-depleted CB mononuclear cells were activated with 100 Gy-irradiated K562-mb15-41BBL cells and cultured in the presence of 20 IU/mL IL-2. After 1 week, a retrovirus carrying the B7-H3 CAR-T2A-IL-15 cDNA was introduced into CB-derived NK cells using RetroNectin. The retronectin concentration was 20 μg/ml. Retroviral tier was 4.5 × 10^6^ cfu/ml. Subsequently, the cells were re-exposed to 100 Gy-irradiated K562-mb15-41BBL cells, cultured for another week, and then prepared for subsequent experiments. The efficiency of CAR transduction was assessed by staining the cells with a goat antihuman F(ab')2 Alexa Fluor 647 antibody.

## Cytokine Release Assays

Cytokine expression by B7-H3 CAR-T or control T cells, co-cultured with U87MG and U87MG B7-H3 KO cells, was evaluated using Quantikine ELISA kits for IL-2 and IFN-γ (R&D Systems Inc., Minneapolis, MN, USA). Effector and target cells, at a 1:1 effector/target (E/T) ratio (1 × 10^5^ each), were co-cultured for 24 h in triplicate wells.

## Cytotoxicity Assay

The ability of CAR-NK cells to lyse tumor cells was determined using the ^51^Cr release assay. Briefly, 1 × 10^6^ target cells were labeled with 200 μCi of [^51^Cr] sodium chromate (GE Healthcare, Chicago, IL, USA) for 2 h at 37 °C. The labeled target cells (1 × 10^4^) were then incubated with effector cells for 4 h at E/T ratios of 0.8, 2.4, and 7.2. The amount of ^51^Cr released in the harvested supernatants was measured using a gamma counter. Maximum and spontaneous ^51^Cr release was ascertained by incubating 1 × 10^4^ labeled targets in 1% Triton X-100 and culture medium, respectively, in triplicate wells. The percentage of specific lysis was calculated using the following formula: [(specific ^51^Cr release − spontaneous ^51^Cr release) / (maximum ^51^Cr release − spontaneous ^51^Cr release)] × 100.

## In Vivo Xenograft Murine Models

We established orthotopic patient-derived xenografts using NOD/Shi-scid IL2Rγ-KO mice (NOG). The mice were anesthetized with isoflurane, after which a skull burr hole was created using a drill. Subsequently, 2 × 10^5^ GDC519 cells, labeled with GFP/luciferase, were injected into the right cerebrum using a stereotactic injector (Muromachi Kikai, Osaka, Japan). The injection site in the cerebrum was located 1 mm anterior to the bregma, 2 mm to the right, and 2 mm deep. Five-day post-tumor injection, tumor engraftment in the head was confirmed using the in vivo imaging system (IVIS) (PerkinElmer Inc., Waltham, MA, USA) following the administration of 150 μL of luciferin (Promega, Madison, WI, USA). Six days after the tumor injections, 2 × 10^6^ B7-H3 CAR-T cells or B7-H3 CAR-NK cells were injected at the same site. Tumor volume was assessed weekly using IVIS, and the mice were ethically euthanized when they displayed neurological symptoms. In the analysis, we randomly assigned mice to the treatment or control group.

## Statistical Analysis

Statistical analysis was conducted using the JMP software (version 16.0; SAS Institute, Cary, NC, USA; RRID: SCR_008567). An unpaired two-tailed Student's *t*-test was utilized for comparisons between groups. Results with P < 0.05 were considered statistically significant.

## Results

### B7-H3 *CAR*-T cells exhibited anti-GBM effect in vitro and in vivo

**Fig. 1:**
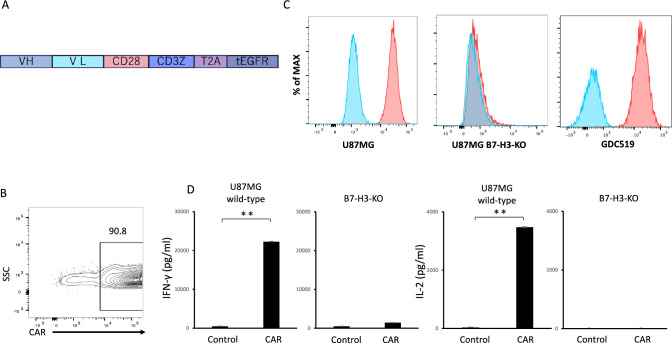
Generation of B7-H3 CAR-T cells. (A) CAR construct targeting B7-H3. VH: variable regions of heavy chain, VL: variable region of light chain. (B) A representative flow cytometric analysis showing B7-H3 CAR transduction efficiency in T cells 7 days after transduction. (C) Flow cytometric analysis of B7-H3 expression in the indicated cells. The blue histogram indicates the isotype control. (D) Secretion of IFN-γ and IL-2 by B7-H3 CAR-T cells or CD19 CAR-T cells (used as a control targeting an irrelevant antigen) after co-culture with either wild-type or B7-H3-knockout (KO) U87MG cells. A representative result from three independent experiments is shown. Data are expressed as mean ± SEM. *P < 0.05 and **P < 0.01, determined by a two-tailed Student’s *t*-test

We constructed B7-H3 CAR consisting of scFv derived from a previously reported anti-B7-H3 monoclonal antibody BRCA84D (MG27A) [33] and the cytoplasmic domains of CD28 and CD3ζ, and transduced the B7-H3 CAR into human T cells (Fig. 1A, B). The B7-H3 CAR-T cells produced IFN-γ and IL-2 when co-cultured with U87MG cells but not when co-cultured with B7-H3-deficient U87MG cells, which were generated using CRISPR-Cas9 (Fig. 1C, D).

**Fig. 2:**
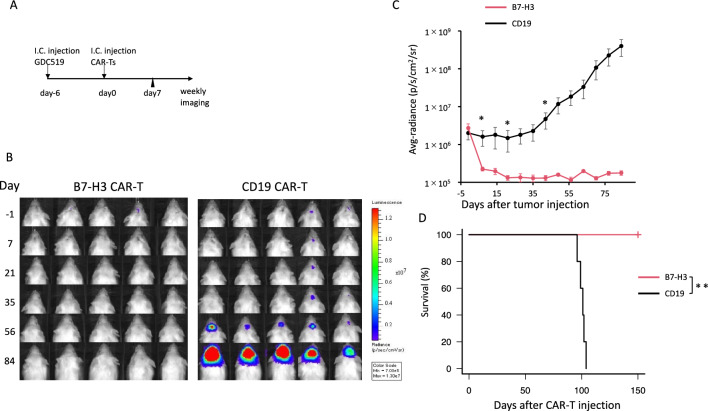
**Antitumor effect of B7-H3 CAR-T cells in vivo.** (A) Experimental design. i.c.: intracranial injection. (B) Bioluminescence imaging of mice treated with either B7-H3 or CD19 CAR-T cells (n = 5 per group). Avg. = average, p = photons; sr = steradian, Min = minimum. (C) Quantification of whole body luminescence. (D) Survival curves of mice treated with either B7-H3 or CD19 CAR-T cells. T cells for these experiments were derived from a single donor. Data are expressed as mean ± SEM. *P < 0.05 and **P < 0.01, determined by a two-tailed Student’s *t*-test (C) and log-rank test (D)

We then evaluated the antitumor effects of B7-H3 CAR-T cells using orthotopic xenografts with GDC519 cells, established from a patient’s GBM tumor cells [9]. We injected GDC519 cells, expressing GFP and luciferase, into the brains of mice (Fig. 2A). After confirming tumor engraftment by IVIS imaging on day 5 following tumor cell injection, we administered B7-H3 CAR-T cells or control CAR-T cells into the brain on day 6 (Fig. 2B). Tumor burden was significantly reduced in mice treated with B7-H3 CAR-T cells compared with that in mice treated with control CAR-T cells (Fig. 2C). The survival of the mice treated with the B7-H3 CAR-T cells was significantly longer than that of mice treated with the control T cell (Fig. 2D). No apparent toxicity was observed in mice treated with the B7-H3 CAR-T cells or control T cells.

## Cord blood-derived NK cells transduced with B7-H3 CAR exerted anti-GBM effect in vitro

**Fig. 3:**
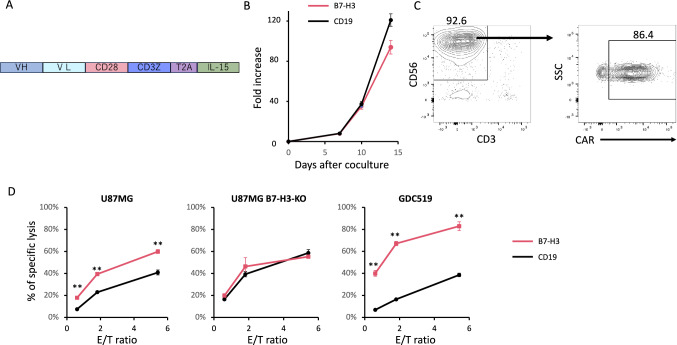
**Generation of B7-H3 CAR-NK cells.** (A) The construct used to generate B7-H3 CAR-NK cells. (B) Growth curve of NK cells transduced with the anti-B7-H3 CAR-NK construct or the CD19 CAR (C) A representative flow cytometric analysis of CD56 and CD3 expression and CAR transduction efficiency in B7-H3 CAR-NK cells 7 days after transduction. (D) ^51^Cr release assay to evaluate specific target cell lysis by the B7-H3 CAR-NK cells versus control CAR-NK cells (CD19 CAR-NK cells), performed in technical triplicates. E/T = effector/target ratio

B7-H3 CAR-NK cells and secreting IL-15 were developed according to the method described in previous studies (Fig. 3A) [17, 23]. Briefly, CB mononuclear cells were stimulated with irradiated K562 cells expressing 4-1BB ligand and membrane-bound IL-15, and cultured in a medium supplemented with IL-2. After 7 days of culture, the cells were retrovirally transduced with B7-H3 CAR-T2A-IL-15 and then restimulated with K562-stimulator cells. After 2 weeks of culture, NK cell expansion was more than 80-fold (Fig. 3B). The efficiency of CAR transduction was over 80% (Fig. 3C). B7-H3 CAR-NK cells exhibited significant cytotoxicity against U87MG cells and the patient-derived GBM cell line GDC519, but not against B7-H3-KO U87MG cells (Fig. 3D).

## Cord blood-derived NK cells transduced with B7-H3 CAR exerted anti-GBM effect in vivo

**Fig. 4:**
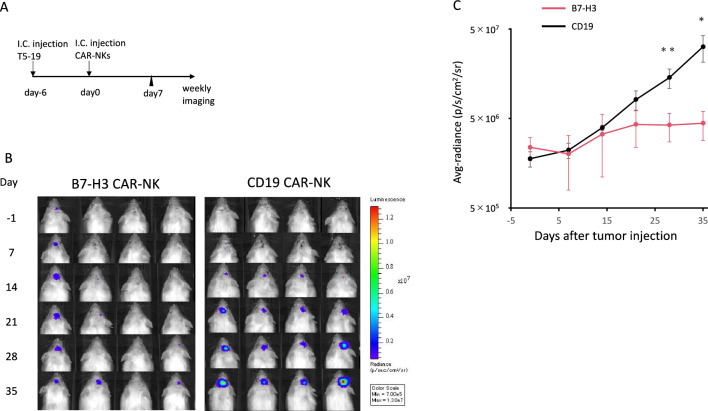
**Antitumor effect of B7-H3 CAR-NK cells in a mouse xenograft model.** (A) Experimental design. (B) Bioluminescence imaging of mice receiving either B7-H3 or CD19 CAR-NK (control) cells. Avg. = average, p = photons, sr = steradian, Min = minimum. (C) Quantification of whole body luminescence. Data are expressed as mean ± SEM. *P < 0.05 and **P < 0.01, determined by a two-tailed Student’s *t*-test

We injected GDC519 cells, expressing GFP and luciferase, into the mouse brain. After confirming tumor engraftment using IVIS imaging on day 5 after tumor cell injection, we administered B7-H3 CAR-NK cells or the control CAR-NK cells into the brain on day 6 (Fig. 4A). Tumor burden was significantly reduced in mice treated with B7-H3 CAR-NK cells compared with that in mice treated with the control CAR-NK cells (Fig. 4B, C).

## Discussion

The efficacy of cord blood CAR-NK cells has been demonstrated in clinical trials against hematological cancers [18, 34, 35]. The anti-GBM efficacy of CAR-NK cells has been previously documented [20–24, 26–28, 36, 37]. B7-H3 has been reported to be a promising target for CAR-T/NK cells [9, 13, 14, 28, 38, 39]. Consistent with these previous reports, in this study we demonstrated that CB-derived B7-H3 CAR-NK cells have the potential to eliminate GBM cells in vivo using a GBM patient-derived xenograft model, although it remains unclear whether treatment with the B7-H3 CAR-NK cells improves the survival of the mice and needs to be clarified in the future. While several previous preclinical experiments have used commercially available GBM cell lines such as U87MG and U251 [21, 26, 27, 37], we used a patient-derived xenograft model, which more accurately reflects the characteristics of patient tumors [40, 41], similar to some previous studies [20, 22, 25, 36].

Although we did not directly compare the efficacy of CAR-T cells and CAR-NK cells targeting B7-H3, the results of the in vivo xenograft model suggested that the effect of CAR-NK cells was modest compared to CAR-T cells. The duration of in vivo persistence of CAR-NK cells has been reported to be shorter than that of CAR-T cells [42], although we did not investigate this. The potential of in vivo persistence of CAR-NK cells needs to be clarified in the future. In addition, more extensive analysis of the phenotypes and biological activities of CAR-NK cells will be required in the future. Furthermore, the toxicity of B7-H3 CAR-NK cells is difficult to study in a xenograft model and will need to be tested in syngeneic GBM models. It may be important to develop methods to improve the antitumor efficacy of CAR-NK cell therapy against GBM. Regulation of the tumor microenvironment is important to enhance the antitumor efficacy of cancer immunotherapy including CAR-T cell therapy [43–45]. For example, the interaction between tumor-associated macrophages (TAMs) and T cells has been suggested by single-cell RNA-seq analysis of GBM samples [46]. Regulation of macrophages and other cell types in the tumor microenvironment will also be important to enhance the efficacy of CAR-NK cells. However, the interaction between CAR-NK cells and other immune cells present in the tumor microenvironment cannot be analyzed in the xenograft model using immunodeficient mice. Future studies using murine CAR-T/NK cells in syngeneic murine GBM models are needed. The limitation of this study is that we performed the CAR-T/NK cell in vivo experiment using only a single donor-derived cells. Future studies using multiple donor cord blood are needed. In addition, we did not analyze the phenotype of NK cells before and after CAR transduction. Since the NK cell population contains several subsets with different potentials [47], the character of B7-H3 CAR-NK cells should be clarified in the future.

In conclusion, CB-derived B7-H3 CAR-NK cells demonstrated an antitumor effect against GBM in a xenograft model generated with patient-derived GBM cells, although more detailed analysis of the B7-H3 CAR-NK cells and their improvement are needed in the future.

## Data Availability

No datasets were generated or analyzed during the current study.

## Abbreviations

CAR: Chimeric antigen receptor
CB: Cord blood
E/T: Effector/target ratio
EGF: Epidermal growth factor
EGFR: Epidermal growth factor receptor
EGFRvIII: Epidermal growth factor receptor variant III
GBM: Glioblastoma multiforme
GVHD: Graft-versus-host disease
HER2: Human epidermal growth factor receptor 2
IVIS In: Vivo Imaging System
IL13Rα2: Interleukin-13 receptor alpha 2
KO: Knockout
NK: Natural killer
NOG: NOD/Shi-scid IL2Rγnull
PBMC: Peripheral blood mononuclear cell ()
RNP: Ribonucleoprotein
scFv: Single-chain variable fragment
